# Insecticide efficacy and emergence timing of the Douglas-fir twig weevil

**DOI:** 10.1093/jee/toae048

**Published:** 2024-03-28

**Authors:** Thomas D Whitney, Gary Chastagner

**Affiliations:** The Davey Tree Expert Company, Davey Institute, 1500 N Mantua Street, Kent, OH 44240, USA; Department of Plant Pathology, Washington State University Puyallup Research and Extension Center, 2606 W Pioneer Avenue, Puyallup, WA 98371, USA; Department of Plant Pathology, Washington State University Puyallup Research and Extension Center, 2606 W Pioneer Avenue, Puyallup, WA 98371, USA

**Keywords:** Christmas trees, export pest, residual toxicity, degree-day model, knockdown

## Abstract

The Douglas-fir twig weevil (*Cylindrocopturus furnissi* Buchanan) (Coleoptera: Curculionidae) has recently emerged as a significant pest of Christmas trees grown in the Pacific Northwest United States. The larvae girdle and disfigure twigs, which adversely affects tree marketability. Trees produced for export are also routinely destroyed for phytosanitary reasons when *C. furnissi* is discovered at border crossings. Due to historically being a sporadic and benign pest on planted and natural Douglas-fir (*Psuedotsuga menziesii*), there is a lack of chemical management options. In laboratory experiments, we assessed the knockdown effects (ability to kill or incapacitate) of 4 insecticides commonly used on Christmas trees: one assay tested knockdown after direct contact for 24 h, and the other assay tested knockdown after being allowed to feed on treated twigs with 2 days, 7 days, and 14 days residuals. Concurrently, we monitored temperature and adult *C. furnissi* emergence at a noble fir bough farm for 2 years to estimate the ideal degree-day window for applying insecticides. Bifenthrin and esfenvalerate knocked down all weevils on contact within just 4 h, whereas chlorpyrifos and acephate failed to achieve 100% knockdown within 24 h. Only acephate failed to knock down more weevils than the control (water) after feeding on treated twigs, regardless of the insecticide residue age. Degree-day modeling revealed a variable emergence window between the 2 years but 50% of adult emergence occurred between approximately 1,000–1,100 degree days (1st January, 50 °F (10 °C), single sine). Future work should assess the resulting management recommendation: apply bifenthrin or esfenvalerate once annually just after 1,000 growing degree days for 2 or more years prior to harvest.

## Introduction

Farm-grown Christmas trees in the United States are a $553 million annual industry, and the Pacific Northwest region, namely Oregon and Washington, accounts for approximately 40% of total trees produced (USDA National Agricultural Statistics Service 2024). Most trees are sold domestically, but the export market is also important. For instance, of the over 5.2 million total trees harvested in Oregon and Washington in 2017, 16% were exported to Mexico (Pacific Northwest Christmas Tree Association 2017). The most common quarantine pests currently intercepted in Pacific Northwest Christmas trees are slugs and snails (Class Gastropoda), yellowjackets (*Vespula* spp.), root weevils (*Otiorhynchus* spp.), the Douglas-fir needle midge (*Contarinia constricta*), and the Douglas-fir twig weevil, *Cylindrocopturus furnissi* Buchanan (Coleoptera: Curculionidae) (Oregon State University Extension 2023).

**Fig. 1.**
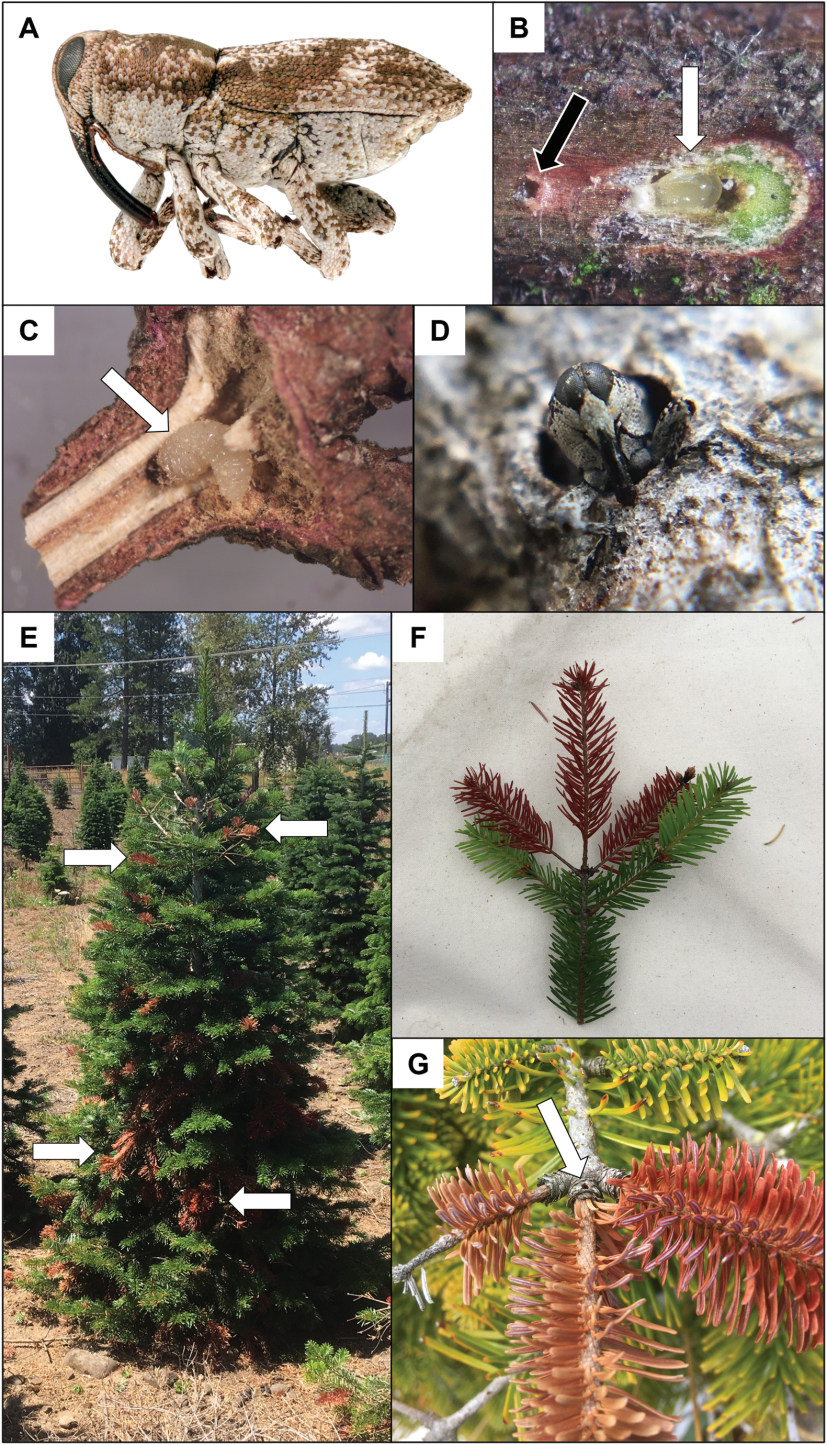
Life cycle and damage caused by A) the Douglas-fir twig weevil, *Cylindrocopturus furnissi*. B) Adults are approximately 3 mm in size and create small holes (black arrow) to feed in the inner bark of the twig. Females lay eggs (white arrow) under bark cuticles, often behind needle scars. C) Larvae feed within nodes and internodal twig vasculature and pith. D) After pupation, adults emerge, completing the 1-year life cycle. Girdling of twigs causes tip dieback of several Christmas tree species, including noble fir (*Abies procera*), E) Nordmann fir (*A. nordmanniana*) and F) Douglas-fir (*Psuedotsuga menziesii*). Dieback can often be attributed to *C. furnissi* infestation by locating the exit hole left behind at the node G). Photo credits: Thomas Shahan, Oregon Department of Agriculture (A), Thomas D. Whitney (B–G).

Native to the Pacific coast of North America, *C. furnissi* is a small beetle whose larvae develop within Douglas-fir (*Psuedotsuga menziesii*) shoots and cause minor twig dieback (Buchanan 1940, Furniss 1942) (Fig. 1). There is one main generation and possibly a partial second generation per year. New adults emerge in summer and feed on the inner bark of small branches during an approximately month-long preoviposition period. Females lay eggs within the living inner bark of twigs starting late summer and into fall, and most will hatch before the dormant season. Larvae (within twigs) and adults (clinging to twigs) both overwinter. Larval feeding occurs longitudinally within twig vascular tissue and eventually within the pith, causing girdling, especially at the internodes. Second- and third-year growth are the most common twig ages for *C. furnissi* larval feeding (Whitney, unpublished data). Trees most susceptible to *C. furnissi* attack are young, open-grown saplings, especially those on marginal soils and/or experiencing drought (Furniss 1942).

Prior to the 2010s, *C. furnissi* had little economic importance. The symptoms it causes are inconsequential in natural systems, and it has historically been considered an infrequent and minor pest for Christmas trees and conifer seedling production (Furniss 1942, Overhulser 1980). Factors such as host resistance, intra-specific competition, and the presence of several parasitoids have likely mitigated significant economic losses (Furniss 1942). Starting in the late 2000s, however, *C. furnissi* incidence has become increasingly common in Douglas-fir Christmas tree production stands in the Pacific Northwest United States (DeFrancesco and Murray 2009). True firs (*Abies* spp.) grown for Christmas trees and boughs are also sustaining severe infestations in the region. Despite no prior indication from the published literature, both noble fir and Nordmann fir appear equally as suitable as Douglas-fir for *C. furnissi* development (Whitney, unpublished data). The recent emergence of this native insect as a serious Christmas tree pest for domestic and export markets, as well as its apparent host expansion, has caused concern for the industry.

**Fig. 2.**
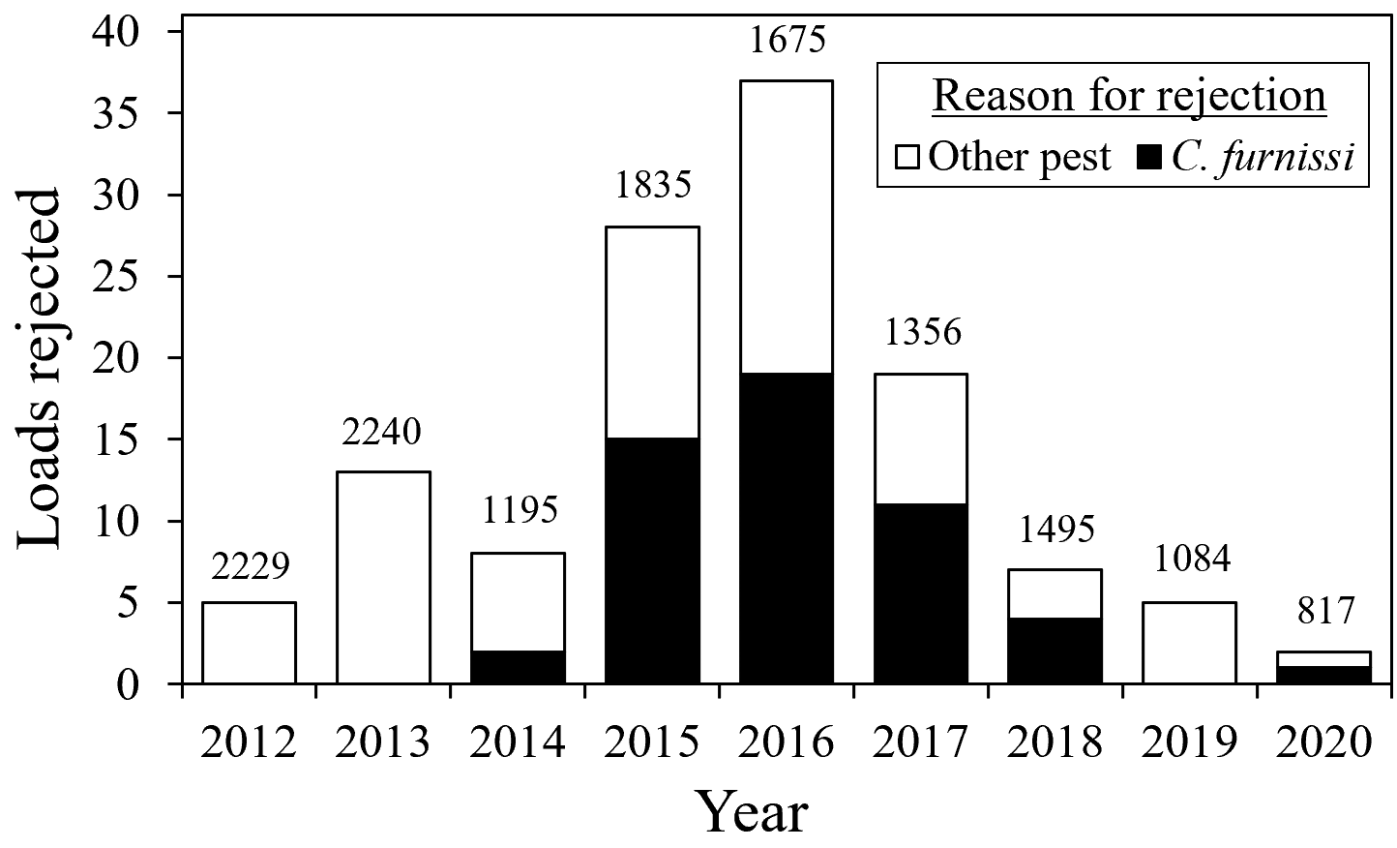
The number of Oregon-produced Christmas tree loads rejected at the Mexican border due to the presence of quarantine pests from 2012 to 2020 (Oregon Department of Agriculture 2013, 2014, 2015, 2016, 2017, 2018, 2019, 2020, 2021). The number of total loads exported to Mexico each year is denoted above the bars. A load is defined as a single truck container usually carrying approximately 500 trees.

Christmas trees have an exceptionally low threshold for aesthetic damage, so the twig girdling and subsequent branch flagging from *C. furnissi* easily reduces tree marketability. Small-sized growers who sell domestically have reported losses of tens of thousands of dollars in a single year (Mark Schmidlin, personal communication). For larger operations, this pest has caused million-dollar losses in the form of rejected export shipments. In 2017 alone, the presence of *C. furnissi* in exported Christmas trees resulted in $2.3 million of losses for Pacific Northwest growers (Dave Silen, personal communication). Mexico instituted a “zero-tolerance” policy for *C. furnissi* in Oregon-grown Christmas trees after recurring interceptions at the border throughout the 2000s and 2010s (DeFrancesco and Murray 2009). In all but 1 year from 2015 to 2020, over 50% of the annual Oregon-grown Christmas tree shipments that Mexico rejected were due to *C. furnissi* (Fig. 2).

Export of Christmas trees and conifer branches out of production regions to domestic or international markets must meet several phytosanitary requirements aimed at reducing the spread of regulated pests and plant pathogens, such as preharvest inspections and specific postharvest management practices (Washington Department of Agriculture 2023). Mexico’s zero tolerance policy for quarantined pests requires that trees be inspected, chemically treated, and subjected to a process of mechanical agitation (Washington Department of Agriculture 2023). For example, they require all imported trees be treated with a pyrethroid insecticide 3–6 wk prior to cutting and undergo mechanical shaking after cutting, a 2-step procedure that effectively reduces yellowjacket infestation risk (Hollingsworth et al. 2009). Although specific pest management recommendations exist for growers to minimize the risk of regulated pests like yellowjackets and slugs, there is no standard for managing or mitigating *C. furnissi*.

Wood-boring insects, such as *Cylindrocopturus* spp., are protected as larvae within the plant vascular tissue from topically applied insecticides. Targeting adults with spray applications before egg-laying can effectively protect trees from wood borers, but success hinges greatly on residual toxicity of the insecticide and timing of application (DeGroot and Helson 1993). Investigation of chemical efficacy (Charlet 1987, Charlet et al. 2007) and life stage phenology (Rogers and Serda 1982, Charlet 1987, Merrill et al. 2010) have led to effective management of the congeneric sunflower stem weevil, *C. adspersus*, on sunflower in the central plains region of the United States (Knodel and Charlet 2002).

This study first compared the contact and residual efficacy of *C. furnissi* adults of 4 broad-spectrum insecticides, 2 organophosphates, and 2 pyrethroids that Christmas tree growers in the Pacific Northwest use regularly. Second, we tracked adult emergence in the field and modeled growing degree days (GDD) to aid in the prediction of ideal timing windows for chemical applications. Our goal was to determine an effective chemical pest management strategy for mitigating *C. furnissi* in Pacific Northwest-grown Christmas trees.

## Materials and Methods

All *C. furnissi* adults used in laboratory experiments were collected from an approximately 17-ha noble fir (*Abies procera*) Christmas bough production farm near Toledo, Washington, USA (46.4386, −122.7585). We utilized a beat sheet method to collect weevils as needed, hitting branches firmly with a wooden stake over a canvas sheet (Bioquip Products Inc., Compton, CA, USA). This site was also used to monitor adult emergence for degree-day modeling.

In 2019, 7 Christmas tree growers in Oregon and Washington were asked about which insecticide products they most use for general pest management. Of the 11 total insecticides that respondents reported, we selected 4 products to test due to their universal popularity and the fact that they are already labeled to control other weevil species (Table 1). Active ingredients (AI) included the organophosphates chlorpyrifos (Lorsban Advanced) and acephate (Orthene 97 Spray), which act to inhibit acetylcholinesterase, as well as the pyrethroids esfenvalerate (Asana XL) and bifenthrin (Brigade 2EC), which act as sodium channel modulators. The knockdown effects of each insecticide, based on direct contact and residual exposure up to 14 days after application, were assessed in 2 experimental assays (as detailed below). A weevil was deemed knocked down if it was dead, incapacitated, or laying on its side or back with legs curled (Baumler and Potter 2007). All statistical analyses were conducted in R version 4.2.2 (R Core Team 2022).

**Table 1.** Insecticides evaluated for knockdown effects against adult *C. furnissi* on Douglas-fir twigs

Active ingredient (AI)	Trade name (formulation)^a^	Percent AI by weight	Application rate (per 1 L water)
Acephate	Orthene Turf, Tree & Ornamental Spray 97	97	0.6 g
Bifenthrin	Brigade 2EC	25.1	1.5 ml
Chlorpyrifos	Lorsban Advanced	40.2	10.1 ml
Esfenvalerate	Asana XL	8.4	0.5 ml

^a^Product sources: Orthene Turf, Tree & Ornamental Spray 97, AMVAC Chemical Corporation, Newport Beach, CA, USA; Brigade 2EC, FMC Corporation, Philadelphia, PA, USA; Lorsban Advanced, Dow Agrosciences LLC, Indianapolis, IN, USA; Asana XL, Valent U.S.A. Corporation, Walnut Creek, CA, USA.

### Contact Toxicity Assay

This experiment sought to determine the toxicity of the insecticides from absorption through the cuticle and not from ingestion. Adult twig weevils were collected in September 2020 and maintained in large mason jars with Douglas-fir twig cuttings for food. Prior to experimentation, individuals were starved for 24 h. Assays were conducted in 45 clear plastic cups (237 ml) maintained at 22.5 °C. Two layers of fine mesh were hot-glued over the openings of lids to promote ventilation and prevent escape, and a 1-cm layer of plaster of Paris was poured into the bottom of each cup to maintain moisture. A single weevil was introduced per cup and left to acclimate for 24 h. We assigned 9 weevils per treatment (4 insecticides + 1 water control) for a total of 45 individuals. Insecticides were mixed at label rates (Table 1) in 50 ml plastic vials under a fume hood. We dipped a cotton swab Q-tip into 1 of the 5 liquid chemical solutions, dabbed the excess moisture on the sides of the vial so that the cotton tip was damp but not wet, and pressed the cotton gently on each weevil’s elytron (dorsal side of the abdomen). New cotton swabs were used for each weevil. Knockdown was monitored at 8 time periods: 1, 2, 3, 4, 5, 6, 7, and 24 h after application.

A logistic regression with repeated measures was used to analyze the interaction between insecticide treatment and the time period of knockdown. “Individual weevil” was treated as a random effect. The data contained instances of complete separation, i.e., none of the weevils were knocked down after the first hour, so we used the bglmer function from package “*blme*” (Chung et al. 2013), which uses a Bayesian a posteriori approach to add a weak, zero mean normal prior on the fixed effects of the model equal to 3 standard deviations. Comparisons of knockdown between insecticides and the control (water) were assessed with Wald tests.

### Residual Toxicity Assay

This experiment sought to determine the toxicity of the insecticides from ingestion of treated twigs at residual ages of up to 2 wk. Insecticide applications were made to 5 identical Douglas-fir clones from a Christmas bough planting grown at the WSU-Puyallup Research and Extension Campus in Puyallup, WA, USA. The trees were 16 years old, healthy, and had been shown previously to be palatable food for *C. furnissi*. Individual branches of the trees were treated at different times in the field and later removed simultaneously for the laboratory experiment. Each of the 4 insecticides and distilled water (control) was mixed within separate hand-held spray bottles at their label rates for application (Table 1). The treatment regime considered each tree to be a block as part of a randomized block design. Fifteen branches on each tree were randomly assigned 1 of the 15 treatment combinations (5 insecticide treatments and 3 application times). Insecticide treatments were sprayed onto the second- and third-year growth segments of branches until thoroughly wet. For each residue age (application conducted 14 days, 7 days, and 2 days before experimentation), this treatment regime was replicated 5 times for a total of 5 trees (block) and 75 branches (experimental unit).

**Fig. 3.**
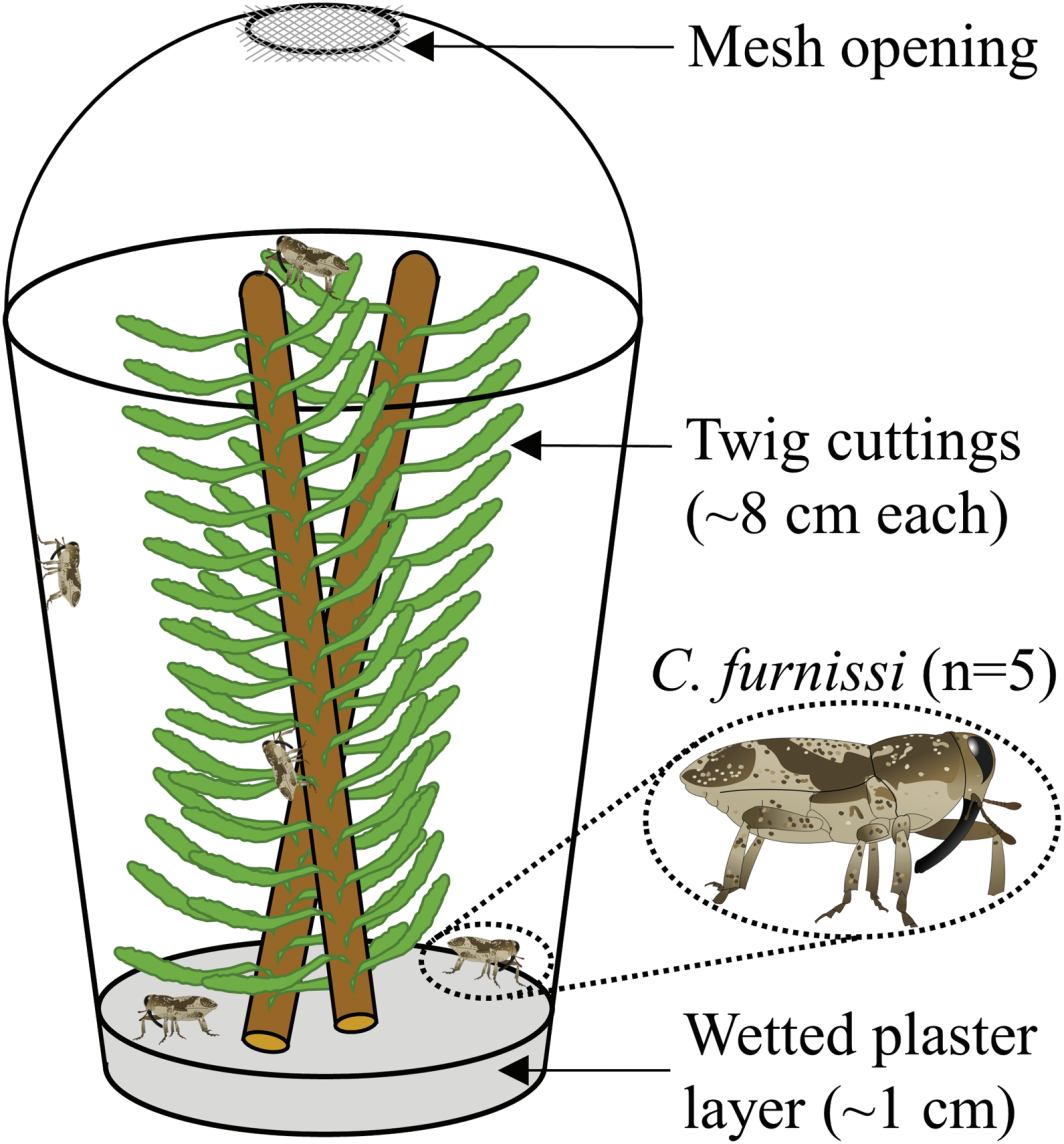
Plastic cup enclosure for residual toxicity assay.

Adult twig weevils were collected in August 2020 and maintained in large mason jars with either noble fir or Douglas fir twig cuttings for food. Prior to experimentation, individuals were starved for 24 h to promote feeding activity on treated twigs. Laboratory assays were conducted in 75 clear plastic cups (355 ml) maintained at 22.5 °C. Two layers of fine mesh were hot-glued over the openings of domed slushie lids to maintain ventilation and prevent escape, and a 1-cm layer of plaster of Paris was poured into the bottom of each cup to maintain moisture (Fig. 3). Five twig weevils were introduced per cup (25 individuals per treatment combination), for a total of 375 individuals, 1 day prior to testing for a 24 h starvation period. On the first day of experimentation, 2 twig cuttings from each treated branch were removed and placed inside the cups: a ~8-cm segment of the second-year growth and a ~8-cm segment of the third-year growth. The weevils were then left to feed for 24 h. After this period (the second day of experimentation), the number of knocked-down weevils in each cup was recorded. In the field, where spray coverage may be imperfect among different trees or parts of single trees, weevils may move among and feed on both treated and untreated twig tissues. To test the extent that weevils can feed and/or recover after exposure to insecticides, the treated twig cuttings were subsequently replaced with untreated, 8-cm, second- and third-year growth cuttings from 1 of the 5 trees collected that morning. Weevils were left to recover, if possible, and feed on untreated twigs for another 24 h. Knockdown was recorded again. Afterward, twig weevil feeding holes were counted on each treated twig and each untreated replacement twig under a stereo microscope.

The number of weevils knocked down per cup was regarded as count data and analyzed using generalized linear models with “tree” as a blocking factor. Separate regressions were conducted for each the treated twigs and the untreated twigs. Additional regressions were conducted for data subsets according to each residual age. The full and subset data violated the assumption of equidispersion, so instead of the Poisson distribution, we used the Conway–Maxwell–Poisson (COM-Poisson) distribution, which includes an extra parameter to account for underdispersion of data (Sellers and Shmueli 2010). Comparisons of knockdown between insecticides and the control (water) were assessed with Wald tests.

Weevil feeding holes on twigs were treated as count or binary data and analyzed using generalized linear models with “tree” as a blocking factor. Similar to the above, separate regressions were conducted on the full treated and untreated twig datasets, as well as data subsets according to residue age. The hypothesis of equidispersion was not rejected for the full datasets, but in certain data subsets there was complete separation, which indicated zero feeding activity for at least one of the treatments. Therefore, the full datasets were analyzed with Poisson regressions as count data and the subset data at each residue age were analyzed with logistic regressions as presence-absence data. We used the logistf function from package “logistf” for logistic regressions with complete separation issues because it utilizes a bias-reduced penalized likelihood logistic regression (Firth 1993, Heinze et al. 2022).

### Degree-Day Modeling

Adult twig weevil exit holes were observed for 2 growing seasons in the noble fir Christmas bough production stand near Toledo, Washington, USA, to model the number of degree days required for adult development. Two plots, each approximately 1 ha in size and 500 m apart, were established for twig monitoring. Hourly temperature was recorded in each plot under the shaded canopy of a tree using a LogTag HAXO-8 logger (LogTag, Auckland, New Zealand) housed within an AcuRite solar radiation shield (Chaney Instrument Co., Lake Geneva, WI, USA) and positioned at 2 feet off the ground. In the springs of 2020 and 2021, respectively, we marked twigs experiencing tip dieback, a common symptom of twig weevil infestation. In the summers of these 2 years, we regularly monitored each of the marked twigs for insect exit holes, a sign of twig weevil adult emergence. Monitoring occurred weekly until peak emergence in June/July when we shifted to a twice or three-times weekly monitoring regime.

Temperature and exit hole data were used to estimate GDD of twig weevil adult emergence with the following parameters: single sine method, base temperature of 50 °F (10 °C), and a default biofix of January 1st. The single sine method, calculated with the software DEGDAY (Higley et al. 1986), assumes the temperature is approximately symmetrical around the maximum temperature, which is a more accurate degree-day methodology in situations where temperature varies above and below the lower threshold temperature for weevil development. Since the developmental temperature range for *C. furnissi* is unknown, we chose 50 °F (10 °C) as the lower temperature threshold with 1st January as the starting date for degree-day accumulation in both 2020 and 2021. These are widely used default values for nonmodel insects (Herms 2004). GDD estimates were used in two 2-parameter logistic regressions using the drm function from the “*drc*” package (Ritz et al. 2015) to model adult emergence that occurred in 2020 and 2021:


$$\begin{array}{l} Y\;\;\;=\frac{1}{1+\exp\left(-b\left(X-e\right)\right)} \end{array}$$

where *Y* = proportion of exit holes observed, *X* = GDD, *b* = slope at inflection point, and *e* = *X* value at inflection point.

## Results

### Contact Toxicity Assay

**Fig. 4.**
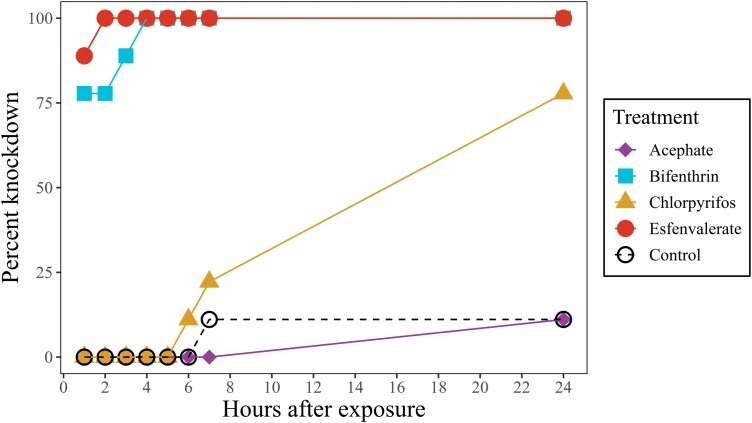
The percentage of *C. furnissi* (*n* = 9) was knocked down after insecticide treatments were applied directly to elytra with Q-tips. Knockdown was assessed at 1, 2, 3, 4, 5, 6, 7, and 24 h after exposure.

Both esfenvalerate (*Z* = 5.34, *df* = 1, *P* < 0.001) and bifenthrin (*Z* = 4.88, *df* = 1, *P* < 0.001) had significantly higher knockdown effects overall than the control treatment. Their effects were rapid, knocking down over 75% of weevils within an hour and knocking down 100% of weevils within 4 h (Fig. 4). In contrast, the knockdown effect of chlorpyrifos was slower, improving significantly between the 1st hour and the 24th hour (*Z* = 2.29, *df* = 1, *P* = 0.02) to 78%. Neither chlorpyrifos (*Z* = -0.25, *df* = 1, *P* = 0.80) nor acephate (*Z* = −1.19, *df* = 1, *P* = 0.23) knocked down significantly more weevils than the control treatment overall.

### Residual Toxicity Assay

**Fig. 5.**
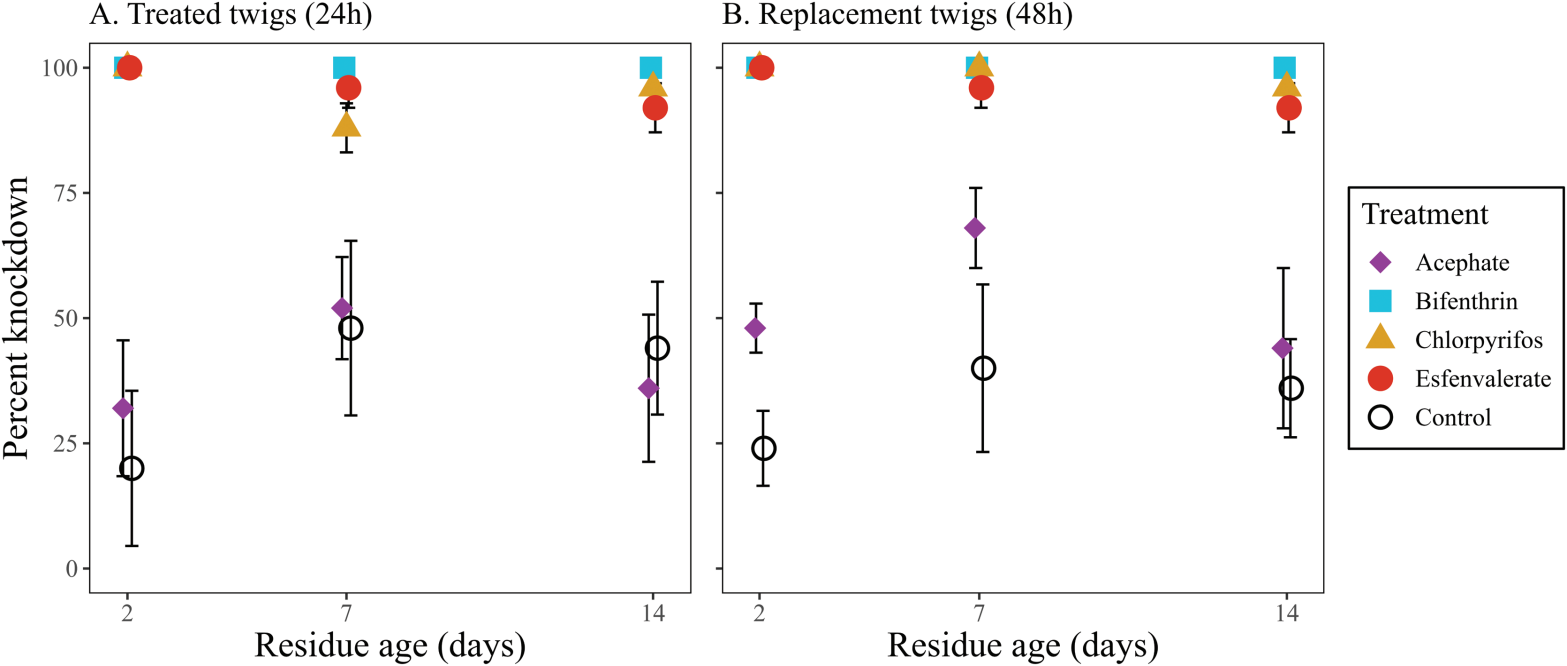
Percentage of *C. furnissi* (*n* = 5 per cohort) knocked down after A) exposure for 24 h to Douglas-fir twigs applied with insecticide treatments 2, 7, and 14 days prior and B) exposure for another 24 h to untreated, replacement Douglas-fir twigs. Knockdown was assessed at 24 h and 48 h. Points on graph utilized a positional dodge to better differentiate error bars.

Weevils were exposed to twigs treated with insecticide of varying residual ages for 24 h (Fig. 5A) and subsequently exposed to untreated twigs for another 24 h to potentially recover (Fig. 5B) to determine if there were differences in knockdown among the 4 insecticides and control (Table 2). Esfenvalerate, bifenthrin, and chlorpyrifos all knocked down significantly more weevils than the control for both treated and untreated twigs and at all residue ages (Table 2). Bifenthrin knocked down 100% of the weevils in all cups. Knockdown of weevils exposed to acephate compared to the control was not significantly different (*Z* = 0.39, *df* = 1, *P* = 0.70), except after 48 h (*Z* = 3.10, *df* = 1, *P* = 0.002) on twigs with 2-day (*Z* = 2.76, *df* = 1, *P* = 0.006) and 7-day residues (*Z* = 2.12, *df* = 1, *P* = 0.03). There was no evidence of weevil recovery on untreated twigs in any insecticide treatment cups.

**Table 2.** Initial knockdown and feeding activity of 5 *C. furnissi* cohorts (each *n* = 5) confined on twigs with different insecticide residue ages, and subsequent knockdown and feeding activity by the same cohorts on untreated twigs the next day

Residue age	Treatment	Initial feeding (24 h) on treated twig		Subsequent feeding (24 h) on untreated twig	
		Mean (± SE) knockdown	Mean (± SE) number of feeding holes	Mean (± SE) knockdown	Mean (± SE) number of feeding holes
2 days	Acephate	1.60 (0.68)	1.00 (0.55)	2.40 (0.24)**	0.60 (0.40)
	Bifenthrin	5.00 (0)**	0.80 (0.37)	5.00 (0)**	0.00 (0)
	Chlorpyrifos	5.00 (0)**	1.20 (0.37)	5.00 (0)**	0.40 (0.24)
	Esfenvalerate	5.00 (0)**	0.00 (0)**	5.00 (0)**	0.40 (0.24)
	Control (water)	1.00 (0.77)	3.60 (0.68)	1.20 (0.37)	0.60 (0.40)
7 days	Acephate	2.60 (0.51)	0.40 (0.24)	3.40 (0.40)*	0.60 (0.40)
	Bifenthrin	5.00 (0)**	0.20 (0.20)	5.00 (0)**	0.25 (0.22)
	Chlorpyrifos	4.40 (0.24)*	1.00 (0.32)	5.00 (0)**	0.20 (0.20)
	Esfenvalerate	4.80 (0.20)*	1.20 (0.97)	4.80 (0.20)**	1.00 (0.37)
	Control (water)	2.40 (0.87)	2.60 (1.21)	2.00 (0.84)	2.00 (1.05)
14 days	Acephate	1.80 (0.73)	2.00 (1.14)	2.20 (0.80)	1.80 (1.32)
	Bifenthrin	5.00 (0)**	0.20 (0.20)	5.00 (0)**	0.00 (0)*
	Chlorpyrifos	4.80 (0.20)*	0.80 (0.49)	4.80 (0.20)**	0.00 (0)*
	Esfenvalerate	4.60 (0.24)*	0.40 (0.40)	4.60 (0.24)**	0.40 (0.40)
	Control (water)	2.20 (0.66)	3.00 (1.10)	1.80 (0.49)	1.60 (0.68)
Total	Acephate	2.00 (0.37)	1.13 (0.43)***	2.67 (0.32)**	1.00 (0.47)
	Bifenthrin	5.00 (0)***	0.40 (0.16)***	5.00 (0)***	0.07 (0.07)**
	Chlorpyrifos	4.73 (0.12)***	1.00 (0.22)***	4.93 (0.07)***	0.20 (0.11)**
	Esfenvalerate	4.80 (0.11)***	0.53 (0.35)***	4.80 (0.11)***	0.57 (0.20)*
	Control (water)	1.87 (0.45)	3.07 (0.56)	1.67 (0.33)	1.40 (0.43)

Within residue age, mean values marked with asterisk (*, **, *** for *P*-values < 0.05, 0.01, and 0.001, respectively) differ significantly from corresponding control.

For analyses of knockdown, COM-Poisson regressions were used.

For analyses of feeding holes, logistic regressions were used within each residue age, and poisson regression was used for the total dataset.

The number of weevil feeding holes on either treated or untreated twigs across treatments ranged from a mean of 0.07–3.07 per cup (Table 2). Significantly fewer holes were observed in cups with weevils exposed to any of the insecticides compared to the control, but within residue ages, there were mostly no differences in the probability that feeding activity occurred in general among the treatments (Table 2).

### Degree-Day Modeling

**Fig. 6.**
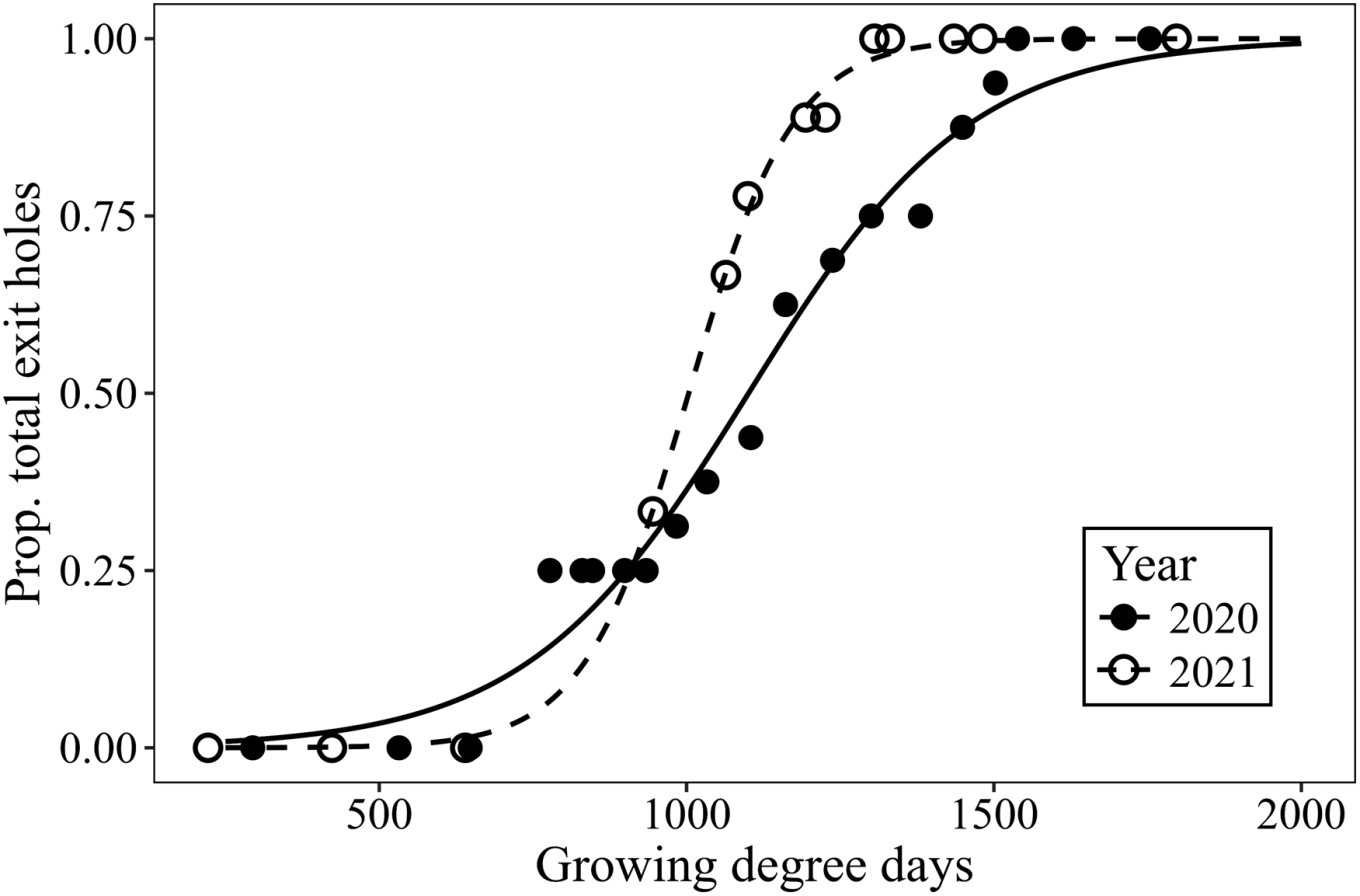
Observed (points) and predicted (lines) growing degree days according to percent *C. furnissi* emergence (i.e., proportion of total exit holes observed) for 2020 and 2021. Degree-day model predictions were generated from 2-parameter logistic regressions.

Sixteen twig weevil exit holes were observed between 25 June and 10 August 2020, and 9 exit holes were observed between 30 June and 20 July 2021. The two-parameter logistic regressions for 2020 (*b* = 0.0055, *e* = 1100.8) and 2021 (*b* = 0.017, *e* = 1003.5) (Fig. 6) were used to estimate the GDD at 10%, 50%, and 90% twig weevil emergence (Table 3). The entire adult twig weevil adult emergence window ranged from an estimated 500–1,500 GDD, with 50% emergence occurring at approximately 1,000–1,100 GDD.

**Table 3.** *Cylindrocopturus furnissi* emergence (estimated from observed exit holes) as predicted from degree-day models in 2020 and 2021

Predicted emergence (%)	GDD^a^ (Date)	
	2020	2021
10	704 (4 July)	816 (26 June)
50	1101 (16 July)	1003 (3 July)
90	1497 (11 August)	1190 (13 July)

^a^GDD = Growing degree days that have accumulated from the model start date of 1st January at a base temperature of 50 °F (10 °C) and calculated with the single sine method.

## Discussion

The 2 pyrethroids tested, bifenthrin and esfenvalerate, were the best-performing AI against *Cylindrocopturus furnissi* adults. Both chemistries knocked down all weevils within 4 h of direct contact and knocked down nearly all weevils after feeding on twigs with 14-day old residue. The residual toxicity of the organophosphate, Chlorpyrifos, was comparably high, but it failed to knock down all weevils within 24 h upon direct contact. Acephate is a widely used organophosphate with systemic activity, commonly used to manage leaf-chewing, sap-feeding, and wood-boring insects, but it was the worst-performing AI against *C. furnissi*. Weevils exposed to acephate on contact showed no ill-effects after 24 h and less successful knockdown than the other insecticides on sprayed twigs.

Adult *C. furnissi* are vulnerable to spray-applied insecticides, whereas larvae and eggs are protected inside twigs. Newly emerged adults require an approximately 1-month preincubation period to feed on twigs and mate before laying eggs (Furniss 1942). Appropriate timing within this window could limit the number of spray applications and maximize the population mitigation effects of insecticides. The total emergence windows we observed varied between years, 47 days in 2020 and 21 days in 2021, but accumulated heat units required for 50% adult emergence only differed by 98 GDD among the 2 years. Three of the 4 tested AI remained potent on twigs after 14 days. Although untested beyond this duration, the pyrethroid formulations we studied likely remain efficacious for much longer than 2 wk—previous work has shown bark applied permethrin (Perm-Up 3.2 EC) on nursery trees to prevent ambrosia beetle attacks for at least 28 days (Reding and Ranger 2018) and bark applied bifenthrin (Onyx) and esfenvalerate (Asana XL) on pines to reduce bark beetle attacks for an entire growing season (Haverty et al. 1998, Fettig et al. 2006).

As further experimentation is being done, we can infer a tentative integrated pest management strategy for *C. furnissi* based on the results from this study and its biological traits. This pest primarily exploits the weakened defenses of stressed trees, so employing preventative measures is advised: avoid planting on droughty sites (or irrigate), avoid planting on sites with poor drainage, and control for weeds that would compete for soil moisture. Additionally, scout for signs and symptoms of *C. furnissi* each spring: branch flagging and associated exit holes (Fig. 1E–G). Insecticide applications are likely warranted when symptoms threaten tree marketability or when growing trees for export markets with zero tolerance for this pest. Within a given year, a single application of a bifenthrin or esfenvalerate formulation at 1,000–1,100 GDD will likely knock down most of the already-emerged weevils on contact before they begin mating and should subsequently knock down most of the remaining weevils once they emerge due to the long-lasting residual toxicity. Complete girdling of individual branches, and therefore flagging symptoms, can sometimes lag behind initial weevil infestation by multiple seasons. Enacting this application protocol annually, starting 2 or more years before harvest, especially for trees grown for export, would likely minimize the incidence of observable damage during the year of harvest and, therefore, also minimize regulatory rejections. Additionally, rather than spraying multiple times a given year, an annual single-spray approach may effectively reduce a *C. furnissi* population while mitigating the risk of secondary pest outbreaks caused by broad-spectrum insecticides (Dutcher 2007).

The degree-day models were derived from limited emergence data at one site on one host species (*Abies procera*) and, therefore, should be treated as a cursory characterization of *C. furnissi* adult emergence timing rather than a robust predictive model. Phenological monitoring of twig boring insects like *Cylindrocopturus* spp. life stages is challenging when hosts are long-lived woody plants. In comparison, monitoring the life stages of the sunflower stem weevil, *C. adspersus*, is easier because sunflowers are annual, low value per individual, and can be destructively sampled (Rogers and Serda 1982, Charlet 1987, Merrill et al. 2010). Conifers grown for Christmas trees, however, are perennial, comparatively high value per individual, and require nondestructive sampling. Christmas trees on largescale production farms are typically sheared once a year during the summer starting 4–5 years prior to harvest, which makes marking and monitoring adult emergence difficult or impossible. Utilization of natural conifer stands would bypass these issues, and in fact, we did monitor *C. furnissi* adult emergence in a natural regeneration Douglas-fir stand in Tayuha State Forest, Washington (47.456304, −122.908894) in 2020. We found similar results based on 8 observed exit holes (emergence: 10% = 623, 50% = 1,025, 90% = 1,428 GDD) but discontinued monitoring this site in 2021 due to storm damage and trail maintenance activities destroying study trees. Therefore, we used a Christmas bough production stand to monitor *C. furnissi* phenology where trees are not sheared and only a small number of 3–4-year-old branches are harvested every fall. Despite the limitations in sample size and site number, our results were informative for developing management recommendations.

The recent emergence of the native *C. furnissi* as a significant pest of Christmas trees in the Pacific Northwest United States presents several questions. Phytosanitary rejections due to *C. furnissi* have increased (Table 2), indicating a potentially higher incidence of infestation across farms in Oregon and Washington. Record-breaking droughts during the 2010s (Parker 2023) may explain this trend because twig weevils, like other wood-boring insects, are often secondary pests that find increased success attacking stressed trees (Ranger et al. 2010). *Cylindrocopturus furnissi* has also seemingly broadened its host breadth to include not only Douglas-fir, *Pseudotsuga menziezii*, but also true firs, *Abies* spp. Whether this novel behavior is a result of recent host-associated differentiation (Hernández-Vera et al. 2010, Forbes et al. 2017, De Biase et al. 2019, Moffat et al. 2019) or rather a result of *C. furnissi* exhibiting a previously undocumented generalist diet due to increased availability of acceptable host choices is unknown. Once the dominant Pacific Northwest-produced Christmas tree species, Douglas fir only represented 50.5% and 36.3% of total harvested trees in 1998 and 2019, respectively, whereas noble fir increased from only 5% of total harvested trees in 1969 to 37.2% in 1998 and 46.8% (62.3% for all true firs) in 2019 (USDA National Agricultural Statistics Service 1998, 2020, Chastagner and Benson 2000). Population genetic analysis of *C. furnissi* collected from different tree species in different locations may elucidate whether any genetic differentiation is associated with host type or geography.

In conclusion, this study revealed that both bifenthrin and esfenvalerate, 2 AI frequently used for broad-spectrum pest suppression in Christmas trees, are effective at knocking down *C. furnissi* adults in laboratory assays. Our phenological monitoring results suggest that one application at approximately 1,000–1,100 GDD should prevent most summer-emerging adults from laying eggs and reinfesting trees, especially if conducted annually multiple years before harvest. Investigating the efficacy of these management recommendations in a field study is warranted, because our results were based on no-choice tests of branches with perfect spray coverage. Also worth exploring is weevil feeding preferences among treated and untreated twigs, as well as the coverage and efficacy of treatments when applied by different application methods typically used in Christmas trees: ground spray equipment or helicopter equipment that sprays aerially.

## Data Availability

Datasets and R code are available at FigShare, DOI: 10.6084/m9.figshare.25122626.
